# Editorial: Genetic and proteomic biomarkers in solid tumor detection and treatment

**DOI:** 10.3389/fgene.2023.1200660

**Published:** 2023-05-11

**Authors:** Jitian Li, Apeng Chen, Ruowen Zhang, Changwei Li, Jianguo Zhou

**Affiliations:** ^1^Laboratory of Molecular Biology, Henan Luoyang Orthopedic Hospital (Henan Provincial Orthopedic Hospital), Zhengzhou, China; ^2^Lanzhou Veterinary Research Institute, Chinese Academy of Agricultural Sciences, Lanzhou, China; ^3^Jiahehongsheng (Shenzhen) Health Industry Group, Shenzhen, China; ^4^School of Medicine, Shanghai Jiao Tong University, Shanghai, China; ^5^University of Erlangen Nuremberg, Erlangen, Germany

**Keywords:** solid tumor, genetic biomarker, proteomic biomarker, detection, treatment

## Introduction

Cancer is a major public health problem worldwide, which has become one of the leading causes of death, and it remains a great burden for the society (Siegel et al., CA Cancer J Clin, 2023, 73, 17–48). That is to say, it is urgent to search for suitable diagnostic, therapeutic, and prognostic methods such as biomarkers to affect the outcome of cancer patients as early as possible. The earlier the diagnosis and treatment, the better the recovery of the patient. An ideal cancer biomarker has high sensitivity and specificity and can reflect the status of the cancer, based on a molecular or process-based change. Biomarkers can help to identify cancer patients at an earlier stage and guide personalized therapy. In recent years, the advances in the genomics, transcriptomics, proteomics, and metabolomics have significantly improved our understanding of cancer biology. Based on these histological techniques, lots of biomarkers of cancer could be screened out and validated, such as nucleic acids, proteins, sugars, small metabolites, and entire tumor cells found in the body fluid, which could be used for diagnosis, treatment efficacy, prognosis, recurrence, and risk assessment (Wu, Chem Soc Rev, 2015, 44, 2963–2997). The evolutionary transformation in personalized cancer therapy and the prognosis of cancer have greatly improved. Recently, molecular biomarkers have played a unique role in cancer management and treatment.

Through research in the field of solid tumor diagnosis and treatment, this Research Topic covers a wide spectrum of the biomarkers from gene to protein. Research from laboratories, results from clinical trials, or reports of interesting cases are within the scope of this current Research Topic. The subtopics include:

1. Identification, characterization, and validation of genetic and proteomic biomarkers that are associated with solid tumor susceptibility, diagnosis, and therapeutics.
2. Novel techniques for identifying or capturing genetic or proteomic biomarkers in solid tumors.
3. Translational research bridging the gap between our incremental knowledge on the association of solid tumor biomarkers and characteristics and the outcome of cancer patients in clinical practice.
4. Case reports of rare cancer types or interesting cases in solid tumors associated with genetic or proteomic biomarkers in clinical practice.

The Research Topic we organized not only included the techniques to identify novel biomarkers and genetic and proteomic biomarkers that are associated with solid tumor susceptibility, diagnosis, and therapeutics, but it also included clinical translational and interesting case reports. Finally, this topic accepted 63 articles, including two brief research reports, one case report, one mini review, one review, one systemic review, and 57 original research articles covering a wide spectrum of studies.

In this Research Topic, many of the studies focused on the identifying, characterizing, and validating of corresponding genetic or proteomic biomarkers associated with tumor detection and therapy. Zhang et al. drew a conclusion that NF1 and NF1-related microRNAs, including hsa-miR-199a-3p and hsa-miR-34a-5p, may be novel biomarkers in diagnosing undifferentiated pleomorphic sarcoma (UPS). Kan et al. identified the hub gene in the tumorigenesis of gastric cancer (GC), obtaining a biomarker prediction model in which the high expression level of ribonuclease P protein subunit p30 (RPP30) was correlated with cancer progression and poor survival, and thus it was considered to be a novel diagnostic and prognostic biomarker. Through bioinformatic analysis, Chen et al. identified the lncRNA/circRNA-miRNA-mRNA ceRNA network as potential diagnostic and prognostic biomarkers in hepatocellular carcinoma (HCC). Luo et al. established a predictive model including three anti-tumor-associated antigen (ENO1, GAPDH, and TPI1) autoantibodies based on SERPA, which could be used as a promising and powerful tool in detecting osteosarcoma (OS). As for esophageal squamous cell carcinoma (ESCC), based on the Gene Expression Profiling Interactive Analysis (GEPIA) platform, Xie et al. concluded that the expression levels of anti-POSTN and anti-TIMP1 autoantibodies were higher in ESCC patients with areas under the ROC (AUC)s of 0.638 and 0.585, respectively, which could distinguish affected patients and non-affected ones, thus acting as potential biomarkers in diagnosing ESCC. Xu et al. showed that PLOD2 plays a significant role in tumorigenesis and maybe serves as a potential biomarker for diagnosis and prognosis through pan-cancer analyses. Early detection of tumors could improve survival, avoiding their progress and lethality as much as possible (Crosby et al., 2022). Sensitive and specific early detection technologies are essential in this research field.

As for the treatment biomarkers, most of the studies chose bioinformatics to screen the potential biomarkers at the first stage and then validated them with experiments, which is a commonly used and convenient research routine. Based on a comprehensive study of bioinformatics and experimental validation, Lin et al. indicated that SPOCD1 might be an independent prognostic factor for ESCC patients in the occurrence and development of the cancer. Li et al. obtained significantly differentially expressed mutant genes between primary and metastatic prostate cancer from the COSMIC database, which showed potential in aiding the treatment strategy in clinical practice. Through the TCGA database, Zhang et al. focused on THBS2, which is related to the poor prognosis and immune infiltration of GC. Wang et al. analyzed the expression of SERPINH1 in the Cancer Genome Atlas and the Genotype-Tissue Expression dataset, after which it was validated and correlated with immune infiltration as a potential prognostic biomarker in a pan-cancer analysis. Based on the bioinformatics analysis and an *in vitro* and *in vivo* study, Zhu et al. indicated that KDF1 plays an important role in ovarian cancer (OC) progression, which might be a therapeutic target for OC patients. Jiang et al. showed that RAB GTPases have an important function in regulating the cell cycle and immune-related pathways based on bioinformatics and functional science, demonstrating their potential as biomarkers in predicting prognosis and immunotherapy response in colorectal cancer (CRC).

A number of studies identified genetic biomarkers for therapy in various solid tumors, including gene mutations, DNA methylation, lncRNAs, miRNAs, and circRNAs. Gao and Shen discussed a common mutation in the KRAS gene, glycine 12 mutated to cysteine (G12C), which could be used in the treatment of non-small-cell lung cancer (NSCLC). Fu et al. explored the association of the *FLG* mutation with tumor mutation load and clinical outcomes, showing that the *FLG* gene mutation might be a protective factor, thus validating its usage as a novel therapeutic target and biomarker for stomach adenocarcinoma (STAD) treatment. As for copy number variations (CNVs), Li et al. identified and validated potential therapeutic and prognostic targets of GC by analyzing single-cell sequencing data, drawing a conclusion that *CPVL* could be a potential prognostic and therapeutic biomarker in GC. One novel pyroptosis-related gene signature consisting of five key DEGs in predicting the prognosis of soft tissue sarcoma was identified and validated, and Qi et al. suggested that it could be an independent prognostic factor and may be an important research direction in future research. Zhu et al. concluded that the regulation of potential candidate gene PAICS (phosphoribosyl aminoimidazole carboxylase, phosphoribosyl aminoimidazole succinocarboxamide synthetase), the metabolic-related gene, is associated with the development and metastasis of OS. Gong et al. discussed the function of PTTG1 in a pan-cancer analysis, which could act as a potential oncogene and associate with immune infiltration, immune checkpoints, tumor mutational burden, and microsatellite instability, indicating that PTTG1 could be a potential biomarker for both the prognosis and outcomes of tumor treatment as well as a promising target in tumor therapy. Polewko-Klim et al. identified four potential drug-targeted genes (ERBB3, ATP7B, ABCC3, and GALNT14) and five drug-related genes for the more precise treatment of esophageal squamous cell carcinoma and adenocarcinoma. The DNA methylation pattern is also a kind of biomarker. Based on 361 breast cancer (BC) incidence-related DNA methylation patterns, Xiong et al. developed a nomogram to quantify the survival probability of BC. A mini review by Zhao and Li concluded that lncRNA DLX6-AS1 could play crucial regulatory roles in various tumors, contributing to pre-clinical therapeutics. Tong et al. constructed a lncRNA-based risk signature including 12 lncRNAs with important prognostic values in predicting lung adenocarcinoma (LUAD). Moreover, they found that NFYC-AS1 and BIRC6 may be potential therapeutic targets. Chen et al. discovered that three necroptosis-related high-risk lncRNAs had the prognostic value of HCC, helping to provide treatments for the patients. Liu et al. built a risk model with 21 signature m7G-related lncRNAs and evaluated the prognosis of colon cancer (CC) patients, showing that this panel could be of help to diagnosis and therapy in the future. Through their analysis of the GEO database, Qiao et al. showed that hsa-miR-557, a kind of miRNA, could inhibit OS growth by modulating the expression of KRAS both *in vivo* and *in vitro*, and it can thus be used as a therapeutic target. Furthermore, circRNAs can also be potential prognostic biomarkers of tumors, such as in renal cell carcinoma (RCC), as analyzed by Liao et al. The metabolism-related genetic biomarker also has the potential to be a therapy target. Dong et al. reported that transcriptome profiles of fatty acid metabolism-related genes are effective for distinguishing cutaneous melanoma (CM) into hot–cold tumors, providing valuable therapy strategies for the effective immunotherapy of patients.

In addition to genetic markers, there are also many studies on various proteomic biomarkers. As proteins are the biological workhorse responsible for most cellular processes, searching for protein biomarkers could be more accurate in reflecting the mechanism of cancer cellular status in disease progression (Tan et al., 2012). There is also plenty of research related to kinds of protein therapy biomarker in this topic. It was concluded by Qin et al. that osteopontin (OPN) is a promising biomarker for cervical cancer, as it acts as a potential therapeutic target involved in immunological activities and multiple tumor processes. Zhang et al. discussed the expression level of anti-silencing function 1B (ASF1B), a histone H3-H4 chaperone, and concluded that it is a promising independent prognostic biomarker and that it may serve as a potential immunotherapeutic target in HCC. Ding et al. drew a conclusion that increased expression of semaphorin 5B (SEMA5B) is associated with immune cell infiltration, and it can be served as a novel diagnostic biomarker and prognostic factor for kidney renal clear cell carcinoma (KIRC). The study by Fang et al. posited that CBX1/3/7/8, a family number of chromobox family proteins (CBXs), could serve as a potential therapeutic target and prognostic biomarker for esophageal carcinoma (ESCA). An inflammation-related signature could also be used as a biomarker in predicting the prognosis of patients. Yu et al. constructed an inflammation-related signature (IRS), which might be sensitive to immune drugs and serve as a biomarker to predict survival in KIRC. Sm proteins (SNRPD1/E/F/G) independently predict the prognostic outcome of LUAD, and Liu et al. explored the function of which ones act as treatment targets. Chemotherapeutic agents can elevate not only the therapeutic effects but also the malignancy of cancer cells. Zhang et al. concluded that through upregulating CNTN-1 in lung adenocarcinoma cells, low-dose cisplatin can activate the epithelial–mesenchymal transition (EMT) process and the resulting malignant progression.

There was also included reported biomarkers about prognosis. Shen et al. discussed the function of NAP1L1 that, through influencing the Wnt/β-Catenin pathway in HCC, may act as a novel prognostic biomarker associated with macrophages. The high expression of Fc receptor-like B (FCRLB) was first reported by Wang et al. to have the function of predicting the poor prognosis of CRC, which could be a potential prognostic biomarker. Hu et al. reported that delta-catenin could attenuate medulloblastoma cell invasion by targeting the EMT pathway, which might be a positive prognostic biomarker. Zhang et al. elucidated that transmembrane protein 170B is a prognostic biomarker, associated with the poor prognosis of pancreatic adenocarcinoma (PAAD), and considered it to be a therapeutic target. As ferroptosis was discovered as a type of regulated cell death recently, the ferroptosis-related gene pair index (FRGPI) was concluded to be an independent prognostic biomarker guiding individualized tumor therapy by Li and Wang. Liu et al. constructed and validated a prognostic model about macrophage differentiation-associated genes (MDGs) to predict the outcomes of clear cell renal cell carcinoma (ccRCC). Zhou et al. developed a gene signature to predict prognosis and resistance in diffuse large B-cell lymphoma (DLBCL), which can not only predict guide individualized treatment but also can predict survival and resistance. Song et al. revealed the important function of histone deacetylases (HDACs) family genes in the efficacy of immunotherapy and chemotherapy of LUAD. Liang et al. identified a novel model consisting of three cancer stem cell-related genes that could accurately and independently predict the clinical outcomes of HCC patients. Xu et al. mainly focused on identifying a novel signature that integrated immunoglobulin (IGHA2), a glycosylation-related gene (SLC35A2), and an anti-viral gene (BST2) as an independent prognostic indicator for BC.

Compared with single biomarkers, a combined panel of biomarkers may have a higher clinical value. The panel established by Luo et al. showed that the sensitivity, specificity, and AUC in diagnosing OS were 70.59%, 86.27%, and 0.798, respectively, higher than the single one. Yang et al. built a new prognostic prediction model based on three ras-related genes with the AUC of 0.932, contributing to providing new insight into both diagnosis and treatment in ESCC. Meng et al. constructed a necroptosis-related miRNA signature consisting of five miRNAs (miR-139-5p, hsa-miR-326, miR-10b-5p, miR-500a-3p, and miR-592) as a powerful tool in predicting the prognosis of HCC of AUC>0.7. A model consisting of liquid–liquid phase separation (LLPS)-correlated genes (LCGs) combined with clinical factors was constructed by Huang et al., who showed that it is a good tool in predicting the prognosis of BC with receiver operating characteristic curve (ROC) values in all cohorts (1/3/5-year ROC values were 0.89, 0.79, and 0.75, respectively).

Novel techniques to identify genetic or proteomic biomarkers were also included. High-throughput sequencing and screening based on identification technologies are particularly suitable for cancer research for diagnosis, prognosis, and therapeutics. Qian et al. detected nine gastrointestinal stromal tumor (GIST)-related gene mutations through targeted next-generation sequencing (NGS), which provide a reference for individualized diagnosis and treatment. The study by Papanikolaou et al. demonstrated that mapping unknown genes to functional pathways by network reconstruction could be used as a powerful tool to identify candidate oncoproteins as biomarkers. Zhong et al. used the CIBERSORT method to establish a prognostic model, revealing that infiltrating immune cells in CRC may be an important determinant of prognosis and immunotherapy. Through single-cell sequencing and machine learning algorithms, Li et al. identified 21 potential essential biomarkers for HCC cells, providing diagnostic and therapeutic value for HCC pathogenesis. Luo et al. used the serological proteome analysis (SERPA) approach to select candidate anti-TAA autoantibodies as biomarkers for OS.

However, a tumor-associated biomarker cannot be applied in a clinical setting until it is confirmed accurate, reproducible, and reliable and has clinical utility (Hayes, 2015). Gallardo-Rincón et al. explored the survival rate of Mexican OC patients with the founder mutation (*BRCA1*-Del ex9-12) after treatment with olaparib. They showed that treatment increased progression-free survival (PFS). A biomarker needs to undergo a lengthy experimental validation process from research to clinical application to accomplish transformation. Only by achieving clinical application can a biomarker truly realize its value.

A case report was contained in this Research Topic. Han et al. presented the diagnosis, tailored genetic counseling, and cancer prevention of a locally advanced lung cancer patient with dMMR/MSI-H/TMB-H tumor and *PMS2*-LS, who received four cycles of nivolumab plus chemotherapy, achieving a disease-free survival of 16 months. Further efforts are required to investigate the efficacy of targeted therapy in rare tumor patients. In a future topic, we hope more case reports of rare cancer types or some other interesting cases in solid tumors associated with biomarkers in clinical practice will be reported for researchers.

The sources of cancer biomarkers are extensive, including blood, urine, feces, cerebrospinal fluid, ascites, pleural fluid, saliva, sweat, skin tissue, oral mucosa, biopsy and puncture tissue, and intraoperative specimens, among others. In this topic, blood is the most common source. Meanwhile, precision medicine acting on a specific target, whether for diagnosis or therapy, highlights the clinical application of biomarkers. However, the occurrence and progression of cancer is complex, which requires more effective and multiple kinds of biomarkers to gain an in-depth understanding of it.

In summary, we are proud of this Research Topic at *Frontiers in Genetics*, which covers many aspects of the biomarkers in solid tumors, especially relating to genetic and proteomic biomarkers, from discovery to clinical application. We hope this Research Topic can provide useful and helpful advice for researchers studying the detection and treatment of solid tumors. Moreover, we thank all authors and reviewers contributed to this Research Topic.
